# Transkingdom interactions between HPV and the microbiome in the female reproductive tract: Gaps, challenges, and emerging perspectives

**DOI:** 10.1371/journal.ppat.1014517

**Published:** 2026-08-19

**Authors:** Chloe Schiff, Paweł Łaniewski, Melissa M. Herbst-Kralovetz, Megan E. Spurgeon

**Affiliations:** 1 John W. and Jeanne M. Rowe Center for Research in Virology, Morgridge Institute for Research, Madison, Wisconsin, United States of America; 2 McArdle Laboratory for Cancer Research, Department of Oncology, University of Wisconsin-Madison School of Medicine and Public Health, Madison, Wisconsin, United States of America; 3 Cellular and Molecular Biology Graduate Program, University of Wisconsin-Madison, Madison, Wisconsin, United States of America; 4 Department of Basic Medical Sciences, College of Medicine-Phoenix, University of Arizona, Phoenix, Arizona, United States of America; 5 University of Arizona Cancer Center, University of Arizona, Tucson/Phoenix, Arizona, United States of America; 6 Department of Obstetrics and Gynecology, College of Medicine-Phoenix, University of Arizona, Phoenix, Arizona, United States of America; 7 University of Wisconsin Carbone Cancer Center, University of Wisconsin–Madison, Madison, Wisconsin, United States of America; University of Iowa, UNITED STATES OF AMERICA

## Introduction

**Fig 1:**
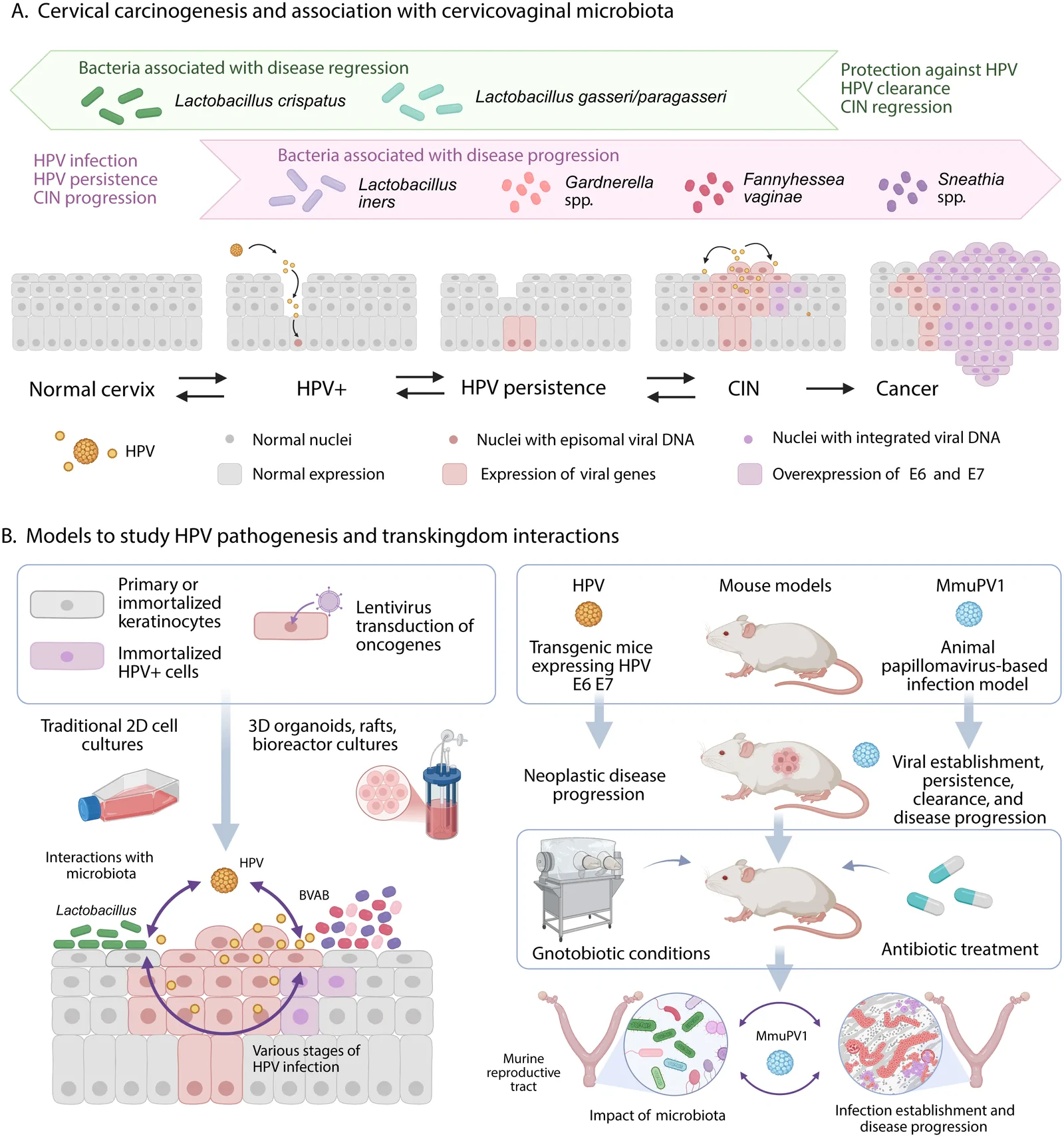
Transkingdom interactions during HPV pathogenesis in the female reproductive tract and preclinical models to define underlying mechanisms. **A)** Overview of HPV-associated disease progression in the cervical stratified squamous epithelium. Prominent stages of this process are depicted and include HPV infection (HPV+), HPV persistence, precancerous lesions or cervical intraepithelial neoplasia (CIN), and cancer. *Lactobacillus* species (spp.) are associated with protection against HPV infection, as well as viral clearance and disease regression (green banner). *Lactobacillus iners, Gardnerella* spp., *Fannyhessea vaginae*, and *Sneathia* spp. are associated with HPV infection, viral persistence, neoplastic disease progression, and cancer development (purple banner). **B)** Overview of preclinical models to identify and study mechanisms of transkingdom interactions. *In vitro* models include 2D and 3D cell culture-based systems (left) and *in vivo* models include HPV transgenic models and animal papillomavirus infection-based murine models (right). Created in BioRender. Herbst-Kralovetz, M. (2026) https://www.BioRender.com/a7jb127.

The composition and dynamics of microorganisms in a host environment, termed the microbiome, can impact fundamental aspects of viral infection and host susceptibility to pathogens. It is increasingly recognized that the complex web of host-microbe-virus interactions ultimately determines the capacity of a virus to cause disease, yet the impact of transkingdom relationships on viral pathogenesis and disease remains understudied for many prominent human pathogens [1]. Human papillomaviruses (HPVs) are the most common sexually transmitted infection (STI) in the US and worldwide and are a prime example of this gap in knowledge [2,3]. Despite the availability of highly effective prophylactic vaccines, high-risk HPV (hrHPV) infections are associated with ~5% of all cancers globally [4]. With a strict tissue tropism for the stratified squamous epithelium, HPVs can infect and occasionally initiate neoplastic progression at both cutaneous surfaces of the skin and the mucosal lining of the upper respiratory tract, anogenital tract, and oral cavity (Fig 1A) [5]. Even though many of these anatomical sites are colonized by well-characterized milieus of microbes, the role of the host microbiome in potentiating and/or limiting HPV pathogenesis and disease has not been fully characterized [6]. In this Pearls review, we discuss the current understanding of the interplay between host bacterial communities and HPVs in the female reproductive tract (FRT), an anatomical site where HPV establishes persistent infections and is a major etiological agent of gynecological cancers. Although the microbiome encompasses many microbes beyond bacteria (including fungi, protists, archaea, and viruses) and future research requires a comprehensive focus on the role of the bacteriome, mycobiome, and virome, we highlight remaining gaps in understanding the relationship between HPV and the bacterial microbiome, focusing on both established and emerging preclinical models to address these outstanding mechanistic questions [7,8].

## Is there a link between the host microbiome, HPV infection, and disease?

Within the FRT, the cervical and vaginal (cervicovaginal) epithelium is colonized by a collection of microorganisms that significantly influences its architecture, physiology, and function [9]. Consequently, the composition of the cervicovaginal microbiome (CVM) contributes to a host microenvironment ranging from permissive to resistant that HPVs and other STIs encounter during infection. Although much of the current framework surrounding the CVM-HPV interplay has originated from clinical and epidemiological findings, preclinical studies in tractable systems are needed to validate hypotheses and further unravel specific mechanisms that may contribute to HPV-mediated disease. The current prevailing model defines an ‘optimal’ CVM as one with low bacterial diversity that is dominated by one or a few *Lactobacillus* species, namely *Lactobacillus crispatus, Lactobacillus gasseri/paragasseri,* or *Lactobacillus jensenii/mulieris.* Lactobacilli maintain mucosal homeostasis and protect against pathogens by maintaining an acidic pH, producing antimicrobial compounds such as lactic acid, and competitively excluding pathogenic microbes by adhering to cervicovaginal mucosa to reinforce epithelial barrier integrity [10,11]. Depletion of *Lactobacillus* spp. in the FRT is associated with increased bacterial diversity and overgrowth of facultative and obligate anaerobic bacteria—often related to a state of microbial dysbiosis known as bacterial vaginosis (BV). Although the exact etiological agent(s) of BV remain unknown, it commonly features cervicovaginal colonization by bacteria within genera such as *Gardnerella*, *Prevotella*, *Sneathia*, and *Fannyhessea,* that may promote local inflammation and disrupt cervicovaginal epithelial barrier function [6,11]. While BV symptoms often include a “fishy” odor and off-white, gray, or greenish vaginal discharge, many patients remain asymptomatic. Remarkably, global surveillance studies estimate that 1 in 4 sexually active individuals with an FRT has BV. An even higher burden (up to 50% prevalence) falls onto resource-limited communities with reduced access to health care [12]. This high prevalence likely reflects that BV-associated bacteria (BVAB) can colonize and be transmitted via both male and female genitalia and are associated with having multiple or new sexual partners as well as inconsistent condom usage [12].

A growing body of evidence demonstrates a significant, yet correlative relationship between CVM composition and HPV pathogenesis (Fig 1A) [13,14]. Infection and neoplastic disease associated with hrHPVs, or those virus genotypes with oncogenic potential, have been consistently associated with lower abundance of *Lactobacillus* species. Notably, *Lactobacillus iners* is an exception to this protective paradigm [13,15,16]. While most *Lactobacillus* spp. produce both D- and L-lactic acid isoforms, *L. iners* lacks the D-lactate dehydrogenase gene, resulting in a perceptible increase in the L:D lactic acid ratio [17]. Several studies have shown that this metabolic shift creates an environment conducive to the invasion of pathogens into the vaginal canal and upper genital tract, although the mechanistic effects of lactic acid isomers on HPV infection and disease progression remain understudied [17]. Depletion of protective *Lactobacillus* spp., increased microbial diversity, and the enrichment of BVAB are also consistently correlated with more severe grades of hrHPV-induced disease such as cervical intraepithelial neoplasia (CIN) and invasive cervical carcinoma [10,16]. Multiple studies have also reported an association between the STI, *Chlamydia trachomatis* and HPV-associated cervical cancer risk, possibly through mechanisms that alter immune cell function [15,18,19].

## What gaps remain in our knowledge about the interplay between HPV and the microbiome?

Although there is a clear association between HPV and the CVM, a historical lack of tractable preclinical papillomavirus infection models has left key mechanistic questions unresolved. For example, it remains unclear whether microbial dysbiosis is a driver or consequence of HPV viral establishment, persistence, and/or neoplastic disease progression. It is possible that microbiota impact host susceptibility through the secretion of bacterial virulence factors such as proteases and sialidases that degrade the protective mucosal barrier as well as metabolites (e.g., lactic acid isomers or short-chain fatty acids) with barrier maintenance, transcriptional regulation, and/or immunomodulatory capabilities [20,21]. Certain BVAB, including *Fusobacterium* and *Sneathia*, have also been shown to produce 2-hydroxyglutarate, an oncometabolite that may work synergistically with HPV oncoproteins (E6 and E7) to promote DNA instability and initiate neoplastic progression [22,23]. Other hallmarks of BV, such as elevated reactive oxygen species and increased apoptosis, may also cooperate with HPV-mediated oncogenesis to accelerate transformation or increase disease severity [22]. It is also possible that CVM dysbiosis and subsequent changes in the tissue microenvironment result in impaired immune cell-mediated control of HPV-positive cells. Alternatively, or in parallel, HPV itself may remodel microbial communities within the CVM to maintain infection and promote disease progression. This framework is supported by the finding that the HPV E7 oncoprotein downregulates expression of host defense peptides that are crucial for the survival and growth of *Lactobacillus* spp. [24]. Together, these data suggest a bidirectional relationship between HPV and the microbiome.

Beyond gaps in understanding mechanisms and causality within the HPV-CVM relationship, the field also lacks insight into how bacterial species are spatially and temporally linked with discrete stages of the HPV life cycle. More specifically, how does the presence of specific BVAB and their secretions or the formation of polymicrobial biofilms (often found in patients with BV and thought to shield pathogenic species from host-protective responses) impact transitions between initial HPV infection, viral persistence, genome integration, oncoprotein expression, and/or progression to cancer? Understanding these spatiotemporal dynamics is crucial for identifying strategies for prevention/interventions and will require the intentional merging of clinical and preclinical approaches. Longitudinal epidemiological studies that integrate complementary sequencing approaches, including 16S rRNA gene profiling and shotgun metagenomics, may help resolve bacterial community structure and function over time while refinement and standardization of patient metadata collection can capture key behavioral, social, and biological variables that shape the microbiome and HPV outcomes. Equally important is the development and use of experimental systems that enable controlled interrogation of microbe-virus-host interactions across defined stages of the HPV life cycle.

## What preclinical models can be used to fill these knowledge gaps?

Studying the HPV-microbiome interplay has historically been limited by the lack of tractable models that recapitulate both the HPV life cycle and the complexity of the microbiome. Recent advances in cell- and animal-based models used to study papillomaviruses and the microbiome will likely potentiate our ability to advance mechanistic understanding of the bidirectional relationship between the microbiome and HPV infection and disease progression.

Various stages of HPV can be studied *in vitro* with traditional 2D cell monolayers or 3D tissue equivalents that mimic the layered architecture of the stratified squamous epithelium (Fig 1B) [25]. Transfecting the HPV genome into primary or immortalized keratinocytes allows for the study of early gene expression while lentivirus transductions of HPV oncoproteins and culturing immortalized HPV-positive cancer cells mimic pre-cancerous and cancerous conditions, respectively. The advent of 3D tissue equivalent models has significantly strengthened HPV research by providing a platform to study differentiation-dependent aspects of the productive HPV life cycle. Enhanced 3D cell culture models using a rotating wall vessel (RWV) bioreactor further advance our ability to replicate host-virus-microbe interactions within the cervicovaginal environment with physiological precision by replicating relevant fluid shearing, sequential colonization of the epithelium by multiple rounds of bacteria, and biofilms [23,26].

*In vitro* models are primed for studying the bidirectional relationship between HPV and the microbiome because they are cost effective and easily manipulable. For example, a recent study found that patient-derived ectocervix organoids genetically modified to express HPV16 E6/E7 can be ‘co-infected’ with *C. trachomatis*—a framework that could be explored with additional pathogenic bacteria and readily extrapolated to other stages of HPV and cancer progression [27]. Maintaining anaerobic bacteria in the aerobic conditions required for epithelial cell culture conditions may be a significant barrier to co-culture models; thus, the use of cell-free supernatants and/or conditioned media can also be harnessed to explore the effect of microbial byproducts on 2D and 3D cultures of HPV-negative and HPV-positive cells [28]. By modulating the timing of microbial colonization in these models of HPV infection and disease progression, we can learn more about the role of specific microbes in promoting or protecting against various aspects of HPV pathogenesis.

Although cell culture is ideal for modeling stages of HPV infection in human cells, small laboratory animal-based models are necessary for studying virus-induced disease in physiologically relevant settings that accurately model the complexity of immune-epithelium-pathogen interactions (Fig 1B). Transgenic mice expressing hrHPV E6 and E7 can be used to model and better understand how the CVM supports neoplastic progression or regression. Indeed, studies in HPV transgenic mice demonstrated that higher grades of cervical dysplasia were associated with increased microbial diversity in the FRT and a downregulation of peptides implicated in maintaining *Lactobacillus* spp. abundance [24]. Due to the strict species specificity of papillomaviruses, animal papillomavirus-based infection models are better suited for studying the complete viral life cycle in a living organism. The recent discovery of a murine papillomavirus, *Mus musculus* papillomavirus 1 (MmuPV1), and the subsequent development of infection-based papillomavirus models in laboratory mice is driving immense progress in understanding notoriously understudied features of papillomavirus pathogenesis, such as viral establishment, clearance, persistence, and progression to cancer [29–31]. Although MmuPV1 exhibits key differences from hrHPVs, most notably the genetic sequences and functions of the E6 and E7 oncogenes, it infects the murine stratified epithelia, is sexually transmitted, and causes cancer at multiple anatomical sites, including the FRT [32–35]. Our experiments have shown that fundamental aspects of MmuPV1 infection, such as viral load, persistence, and disease severity, not only influence both global and local CVM composition, but are also heavily impacted by the bacterial species present at the time of infection [36]. Although a murine model of MmuPV1 allows us to explore the complexity of virus-bacteria interactions in a host environment, it should be noted that human and mice have fundamental differences in the microbial community structure and bacterial species that colonize the FRT. Future studies in antibiotic-treated and/or germ-free mice involving cervicovaginal colonization with single species or “cocktails” of bacteria will provide a platform for mechanistic studies that provide insight into the role of human-relevant bacteria on papillomavirus pathogenesis. When performing such studies, we stress the need to consider immunological consequences of microbiome manipulations [37]. Finally, we emphasize that no model alone holds all the answers to deciphering the HPV-microbiome relationship. A combinatorial approach will be key to advancing our knowledge of HPV molecular virology and for the development of therapeutic interventions.

## What are future therapeutic opportunities for HPV infection and disease?

Given the likely inextricable link between HPV and the CVM, microorganism-based treatment strategies offer an exciting avenue for both prophylactic and therapeutic interventions across all stages of HPV-mediated disease progression. There is currently a broad range of products that target the microbiome. Over the counter (OTC) probiotics support general wellbeing and undergo minimal testing for therapeutic efficacy, while live biotherapeutic products (LBPs) are rationally designed, require rigorous preclinical and clinical testing, and must achieve FDA approval [38]. Many ongoing LBP trials aim to restore *Lactobacillus* dominance following BV treatment to prevent recurrent infections or acquisition of new STIs. Considering lactobacilli are strongly associated with healthy CVMs, this intervention holds substantial promise for treating persistent HPV infections and preventing HPV-associated cancer. Two recent clinical trials demonstrated that vaginal delivery of an active LBP containing a single *Lactobacillus* sp. (LACTIN-V) or a cocktail of multiple *Lactobacillus* spp. (VIBRANT), was associated with decreased vaginal inflammation and restoration of *Lactobacillus* dominance in the vaginal microbiome for at least 10 weeks following treatment, respectively [39,40]. Despite these encouraging results, further interrogation of pre-LBP treatment plans (e.g., antibiotics and/or antimicrobial washes) along with optimization of LBP dose, route, frequency, and timing will be necessary to increase the reproducible efficacy of these therapeutic strategies.

Expanding our mechanistic understanding of the association between CVM and HPV will allow further development of novel antimicrobial interventions to restore vaginal health and promote viral clearance and neoplastic disease regression. This may include OTC vaginal suppositories or topical creams, gels, or films that contain antimicrobial peptides, biofilm disruptors, antifungal treatments, and therapeutic postbiotics. Due to the inherent complexity of the microbiome, patients often experience variable responses to LBPs and OTC interventions—occasionally leading to BV recurrence or antibiotic resistance [39–41]. Thus, we stress the importance of exploring and employing parallel and/or sequential treatment strategies to ensure lasting restoration of cervicovaginal health. Notably, treatment of male genital partners was recently demonstrated to reduce BV recurrence by more than 50%, underscoring the potential role of sexual partner reservoirs in reintroducing bacteria that promote microbial dysbiosis [42]. Therefore, partner-directed interventions with microbiome-based therapeutics may improve and expand HPV treatment options. Live microorganism therapeutics should also be considered for the treatment/prevention of HPV infection and associated disease at other anatomical sites where HPV causes disease (i.e., skin, oropharyngeal cavity, and other anogenital sites).

## Conclusion

Here, we summarize current knowledge of the relationship between HPV pathogenesis and the microbiome within the FRT and outline strategies to advance the field through mechanistic studies in established and emerging preclinical models. Given the inherent complexity of transkingdom interactions, mechanistically defining how microbial communities confer host protection or promote HPV pathogenesis will require integrated approaches that use clinical studies alongside *in vivo* and *in vitro* models. Notably, many of the conceptual and technical challenges highlighted in this review extend beyond the FRT, as microbial contributions to HPV infection and neoplastic progression at other anatomical sites remain poorly understood. Moving forward, the models highlighted here provide a critical foundation to establish causal mechanisms within and beyond the FRT and can also serve as platforms to further evaluate emerging therapeutic interventions to prevent or reduce HPV infection and HPV-associated cancers.
